# The characteristics of the chloroplast genome of the *Michelia martini*

**DOI:** 10.1080/23802359.2020.1788450

**Published:** 2020-07-09

**Authors:** Yanyan Li, Junqing Wang, Peng Li, Huan Wang

**Affiliations:** Pingdingshan University, Pingdingshan, Henan, China

**Keywords:** *Michelia martini*, chloroplast genome, Magnoliaceae, phylogenetic analysis

## Abstract

The chloroplast genome sequence of *Michelia martini* was sequenced using high-throughput sequencing technology and analyzed phylogenetically in the present study. The complete chloroplast genome was 159,819 bp in length, including a large single copy region (LSC) of 88,078 bp and small single copy region (SSC) of 18,801 bp, and a pair of inverted repeat regions (IR) of 26,470 bp. The contents of CG in the chloroplast genome were 39.29%. The sequence contained 128 unique genes, including 81 protein-coding genes, 38 tRNA genes and 8 rRNA genes. The phylogenetic analysis revealed that *M. martini* is closely related to *Magnolia maudiae*.

*Michelia martini* Finet & Gagnepain ex H. Léveillé is an excellent broad-leaved tree species for landscaping and wood production belonging to the Magnoliaceae family. The Magnoliaceae is considered as one of the most primitive groups of angiosperms (Li and Guo 2014). Magnolioideae is thought to include one genus Magnolia (Nooteboom 2000; Figlar and Nooteboom 2004) or divided into several smaller genera (Law 1984; Liu 2004; Xia et al. 2009). The flowering period is from February to march, and the fruit period is from September to October, it also had certain medicinal value of the essential oil from fresh leaves which had significant effects on lowering blood pressure, cough suppression, expectorant and antifungal effects (Lei et al. 2016). However, there has been no genomic studies on *M. martini*. Chloroplast has been a valuable tool to be used for phylogenetic studies due to its gene conservation and the lack of recombination (Ravi et al. 2008; Lin et al. 2012). Here, the complete cp genome sequence of *M. martini* was assembled and analyzed using high-throughput sequencing technology, the complete cp genome (GenBank accession number MT44441) to retrieve valuable resources for the conservation and phylogeny of *M. martini*.

The fresh leaves were collected from three adult *M. martini* plants from that were growing in Longzhong Botanical Garden (32°10′N, 112°10′E), Hubei, China. The specimen of *M. martini* was stored in the herbarium from the Three Gorges University, the accession number was 00126151. The leaves were stored at −80 °C until used. Total DNA was extracted to construct a library for sequencing with Illumina Hiseq 2500 platform (Illumina, San Diego, CA, USA). Additionally, MITObim v 1.8 (https://github.com/chrishah/MITObim) was used to assemble the complete circular cp genome sequence (Hahn et al. 2013). The cp genome was annotated and manually adjusted with CpGAVAS (Liu et al. 2012). the circular plastid genome map was completed with the help of the online program OrganellarGenome DRAW (OGDRAW) (Lohse et al. 2013) and the annotated sequence was submitted to NCBI.

The total length of the chloroplast genome was 159,819 bp presenting a typical quadripartite structure, of which the length of a large single copy region (LSC) was 88,078 bp and the length of a small single copy (SSC) region was 18,801 bp, which were separated by the IRA and IRB of 26,470 bp. The contents of CG in the chloroplast genome was 39.29%. A total of 128 chloroplast genes were annotated, containing 81 protein-coding genes (63.28%), 38 transporter RNA genes (29.69%), eight ribosomal RNA genes (6.55%) , one pseudogene (0.78%) was inferred to be pseudogenes. Ten protein-coding genes (*rps12*, *rpl2*, *trnV-UAC*, *trnL-UAA*, *atpF*, *ycf68*, *trnI-GAU*, *ndhA*, *trnA-UGC* and *ndhB*) contained one intron, while *clpP* and *ycf3* each contained two introns.

A characterization of the genetic relationship between *M. martini* and related taxa may provide valuable information for broadening the genetic basis of rootstock breeding programs. Thus, we further investigated the phylogenetic relationships between the chloroplast genome sequences of *M. martini* and 19 other species of Magnoliaceae reported in Genbank of NCBI database using MEGA 7.0 (Kumar et al. 2016) (https://www.megasoftware.net). The phylogenetic analysis showed that *M. martini* was closely related with *Magnolia maudiae*, forming a clade included in *Magnolia* (Figure 1). The cp genome of *M. martini* will provide useful genomic resources for further study on genetic diversity and conservation of this species.

**Figure 1.**
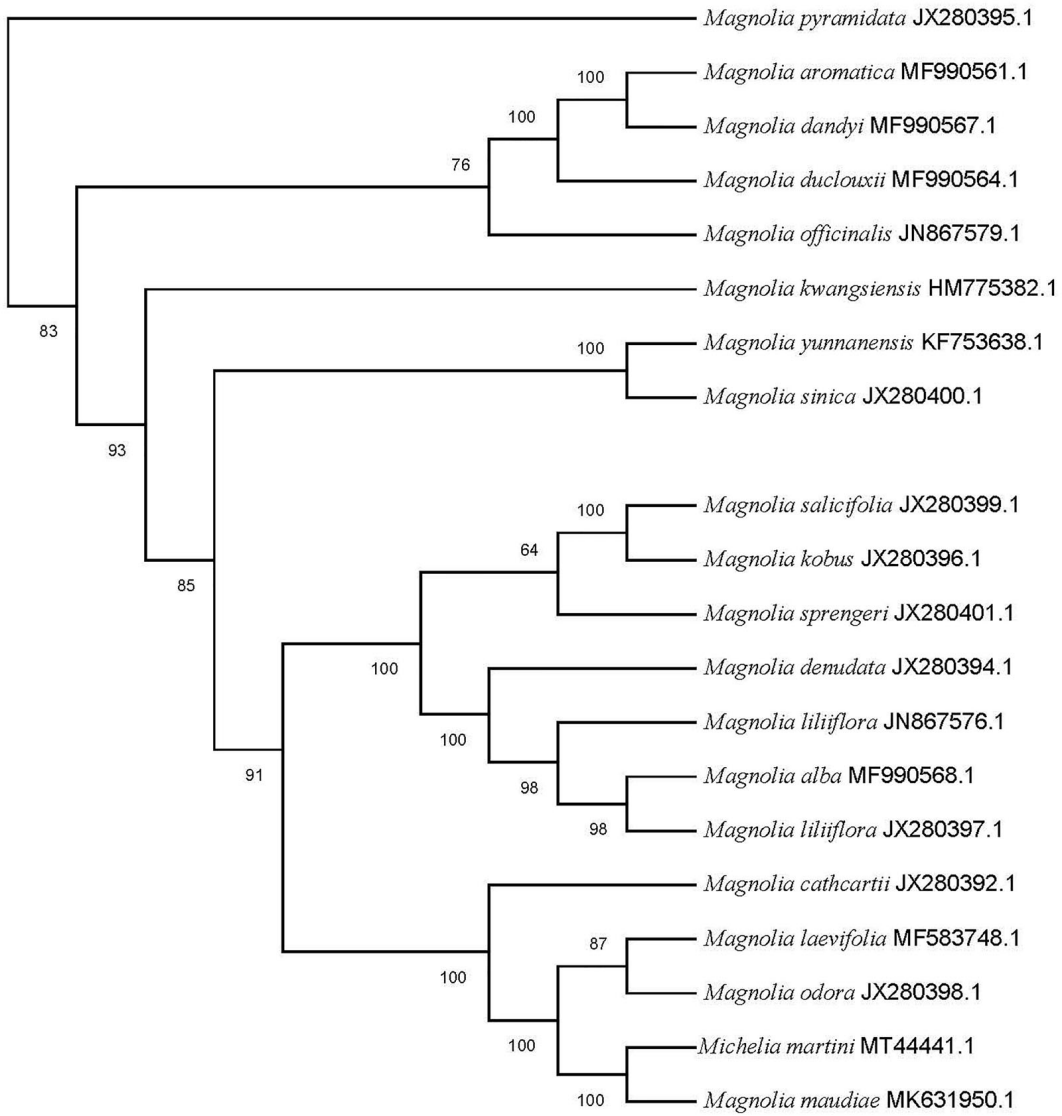
Maximum likelihood phylogenetic tree of 20 selected Magnoliaceae chloroplast genome sequences. Bootstraps (1000 replicates) are shown at the nodes.

## Data Availability

The data that support the findings of this study are openly available in the National Center for Biotechnology Information (NCBI) at https://www.ncbi.nlm.nih.gov/, reference number MT44441.
